# Lattice-Match Stabilization and Magnetic Properties of Metastable Epitaxial Permalloy-Disilicide Nanostructures on a Vicinal Si(111) Substrate

**DOI:** 10.3390/nano11051310

**Published:** 2021-05-16

**Authors:** Anjan Bhukta, Dror Horvitz, Amit Kohn, Ilan Goldfarb

**Affiliations:** 1 Department of Materials Science and Engineering, The Iby and Aladar Fleischman Faculty of Engineering, Tel Aviv University, Ramat Aviv, Tel Aviv 6997801, Israel; anjanbhukta@mail.tau.ac.il (A.B.); akohn@tauex.tau.ac.il (A.K.); 2 Thermo Fisher Scientific, Tel Aviv 7019900, Israel; dror.horvitz@thermofisher.com

**Keywords:** γ-(FeNi)Si_2_ nanoisland, epitaxial silicide, superparamagnetism, scanning tunneling microscopy

## Abstract

We report the epitaxial formation of metastable γ-(Fe_x_Ni_1−x_)Si_2_ nanostructure arrays resulting from the reaction of Ni_80_Fe_20_ permalloy with vicinal Si(111) surface atoms. We then explore the effect of structure and composition on the nanostructure’s magnetic properties. The low-temperature annealing (*T* < 600 °C) of a pre-deposited permalloy film led to solid-phase epitaxial nucleation of compact disk-shaped island nanostructures decorating <110> ledges of the stepped surface, with either (2 × 2) or ($\sqrt{3}\times \sqrt{3}$) R30° reconstructed flat top faces. High resolution scanning transmission electron microscopy analysis demonstrated fully coherent epitaxy of the islands with respect to the substrate, consistent with a well-matched CaF_2_-prototype structure associated with γ-FeSi_2_, along perfect atomically sharp interfaces. Energy dispersive spectroscopy detected ternary composition of the islands, with Fe and Ni atoms confined to the islands, and no trace of segregation. Our magnetometry measurements revealed the superparamagnetic behavior of the silicide islands, with a blocking temperature around 30 K, reflecting the size, shape, and dilute arrangement of the islands in the assembly.

## 1. Introduction

Further technological progress in the field of magnetic data storage and spintronic applications requires the nanoscale miniaturization of magnetic devices with large remanence and energy production [1,2]. This demand has led researchers to combine magnetically hard and soft phases in bi-component type nanomagnets, to facilitate exchange coupling, in the form of core-shell [3,4], bilayer [5,6,7] and nanocomposite [8] structures. In the absence of exchange coupling in single component nanomagnets, remanent magnetic moment, coercive field, and the shape of the magnetization reversal loop, reflect a single magnetically hard or soft phase.

**Figure 1:**
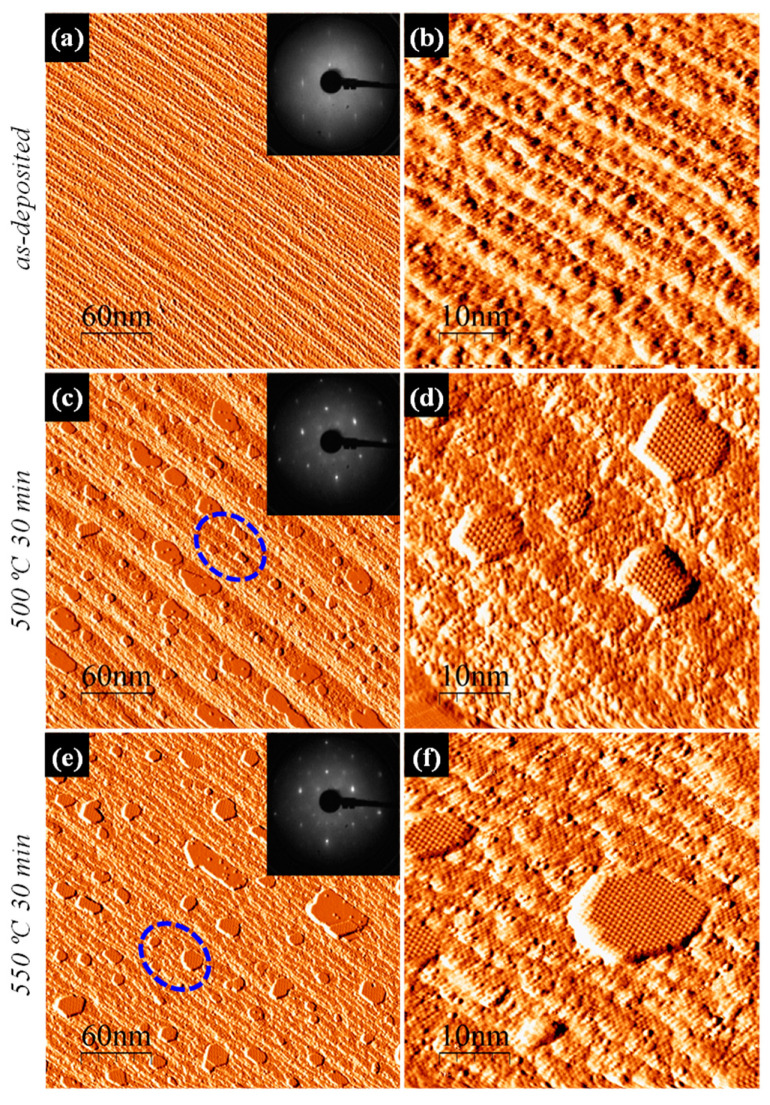
(**a**,**c**,**e**) Low and (**b**,**d**,**f**) high magnification STM topographic derivative images with corresponding LEED patterns in the insets, of various stages of Py SPE growth and anneals on 5°-miscut vicinal Si(111) substrate: (**a**,**b**) as-deposited, and (**c**,**d**) subsequently annealed at 500 °C for 30 min and (**e**,**f**) 550 °C for 30 min. Regions encircled in (**c**,**e**) are the ones blown-up in (**d**,**f**), respectively.

Although many materials in their bulk form exhibit non-ferromagnetic behavior, noticeable ferromagnetic (FM) properties can be observed in nanometric size crystals, where the appearance of uncompensated magnetic moments is attributed to defects, including nanostructure (NS) boundaries [9,10,11]. Specifically in fluorite, R- and F-center defects and their immediate surroundings have been theoretically predicted to produce uncompensated spin moments [12]. Geometric and magnetic anisotropy energy (MAE) of such nanoparticles scales with its size, and aligns its individual magnetic moments into a superspin along a particular direction. When nanoparticles are reduced below single domain size, but above the blocking temperature, the superspin flips back and forth on a time scale shorter than the measurement time, exhibiting zero net magnetic moment, namely superparamagnetism (SPM). SPM refers to dilute, noninteracting assemblies of nanoparticles smaller than a single domain size. In densely packed arrays, interparticle interaction via dipolar fields may take place, leading to superspin glass (SSG) or superferromagnetic (SFM) state, [13,14,15]. These three phenomena of “Supermagnetism” (SPM, SSG and SFM), were previously reported by us to take place in epitaxially self-assembled silicide NSs of transition and rare-earth metals, of nonferromagnetic bulk phase origins [16,17,18,19,20,21]. Often, at least one-dimensional (1D) in-plane ordering of self-assembled silicide phase NSs can be achieved by their decoration of the periodic vicinal Si(hkl) surface step bunches [22,23], as shown in Figure 1 for 5°-miscut Si(111) in this work. 

While defect-induced supermagnetism can be considered “extrinsic”, composition, crystal structure, and the resulting electronic structure of binary (or higher) compounds may still have a sizeable “intrinsic” effect on the overall magnetic properties of compound NSs [21]. Metal silicides usually exhibit a sequence of equilibrium phases and epitaxially-stabilized metastable structures. Depending on deposition method, coverage and heat treatment for Fe−Si reaction, multiple silicide phases and their metastable variants viz. *α*, *β*, *γ*, *s*, have been reported, with miscellaneous structural and physical properties [21]. *α-*phase crystallizes in a tetragonal structure and is metallic, whereas the *β*-phase has an orthorhombic crystal structure and is semiconducting with a direct 0.87 eV band gap, suitable for application in optical fiber communication, thermoelectricity and optoelectronics. The other two often reported structures, i.e., *γ* and *s*, are metastable, with metallic CaF_2_ and CsCl crystal structures, respectively [24,25,26]. In particular, the *γ* structure is predicted to be ferromagnetic, based on band-structure calculations [27]. Metastable *γ* becomes stabilized by favorable lattice match conditions in epitaxial nanoislands on Si substrates, and its magnetic properties have been occasionally explored by analyzing in-plane magnetization reversal loops [17,24,28,29,30]. A kinetic phase diagram, made on the basis of the contact reaction of Fe (with thickness less than 1.5 nm) on Si(111), encompassing the *s* → *γ* → *β* phase transformation sequence for iron disilicide, reported the existence of the *s*-phase in the low temperature regime of 300–400 °C and *γ*-phase in the 470–630 °C range [31,32]. Liang et al. has demonstrated transformation of *s* → *β* nanowires on Si(110) by annealing at 800 °C [33]. Geng et al. [34], using a first principle study based on density functional theory, have explored the effects of epitaxial strain, free surface, interface and edges of grown nanoislands on the resulting magnetic properties, and concluded that only the edges constituted a sizable contribution to the magnetism of γ-FeSi_2_/Si(111) system. By assigning bulk CaF_2_ structure to the γ-FeSi_2_ NSs, their research studied the implicitly combined intrinsic (composition and crystal and electronic structure) and extrinsic (island boundaries) factors.

To understand the relative contributions of intrinsic vs. extrinsic properties to the observed magnetic response, we examined the epitaxial growth of the magnetic alloys (rather than pure metals) on various Si substrates. Permalloy (Py) Ni_80_Fe_20_ was chosen, because it is a commercial soft magnetic alloy, whose magnetic properties are widely studied and well known, and hence easy to compare with those of the epitaxial NSs of its silicide derivatives. Recently, we reported solid phase epitaxial growth from Py Ni_80_Fe_20_ on vicinal Si(111) substrate after a series of stepwise annealing treatments up to 625 °C. In that process, epitaxial disk-like NSs with (2 × 2) reconstructed top facets preceded the bar-shaped *β*-Fe(Ni)Si_2_ ones, with transformation temperature around 625 °C. Annealing at this temperature caused vertical compositional inhomogeneity within the *β*-Fe(Ni)Si_2_ NSs, in terms of different Fe/Ni ratio at the NSs top and bottom parts, whose coupling effectively created an “exchange spring magnet” (ESM) phenomenon [35]. The purpose of the present work was to achieve control over the appearance of different silicide phases and their composition. Specifically, our aim was to stabilize the precursor flat-top ternary γ-(Fe_x_Ni_1−x_)Si_2_ disk-like NSs and avoid the compositional top-to-bottom gradient of Ni and/or its loss altogether. We report here on the successful growth of such γ-(Fe_x_Ni_1−x_)Si_2_ NS disks with (2 × 2) and ($\sqrt{3}\times \sqrt{3}$) R30° reconstructed top facets and uniform ternary composition. Though these γ nanodisks (NDs) were 1D-self-ordered along the periodic ledges of the 5°-miscut vicinal Si(111) substrate by step-decoration mechanism [22,23], as planned, the inter-disk separation distances along the ledge were too large for meaningful dipolar interactions, which accounts for their SPM with a median blocking temperature at around 30 K. 

## 2. Methods

In vacuo part of substrate preparation and in situ measurements were conducted in an ultra-high vacuum (UHV, base pressure ~2.0 × 10^−10^ mbar) variable temperature scanning tunneling microscopy (STM) by Omicron Nanotechnology GmbH, equipped with reflection high energy electron diffraction (RHEED) and 4-grid low energy electron diffraction (LEED)/Auger spectrometer. 10 mm × 1 mm stripes were cut from 5°-miscut Si(111) wafer and ex vacuo chemically degreased prior to introduction into the UHV, where after thorough degassing at ~550 °C for 12–15 h, the sample was repeatedly flashed at 1150–1200 °C to remove the native silicon oxide layer. Consequently, the sample was slowly cooled down to room temperature (RT) to obtain well-ordered (7 × 7) reconstructed terraces separated by periodically bunched steps, evident from electron diffraction and STM measurements. Thin Py film was e-beam evaporated from a high-purity commercial Ni_80_Fe_20_ Py wire onto a flashed stepped Si(111) surface inside the STM stage. A separate sample with an as-deposited Py film was prepared to be used as a reference standard for ex situ chemical and magnetic analyses, with this thin film standard itself calibrated using a bulk piece of the original Py evaporant rod as an absolute standard. Using this rigorous double-standard routine, our X-ray photoelectron spectroscopy (XPS) analysis quantitatively confirmed preservation of the Ni_80_Fe_20_ stoichiometry in this reference film. For LEED study, electron beam energy in the 72–120 eV range was employed. STM images were acquired using the 0.1 nA < *I* < 1.0 nA and −3.25 V < *V* < 3.0 V tunneling current and bias, respectively, in a constant-current mode. STM images were processed using WSxM freeware and commercially available SPIP software from Image Metrology. 

Ex situ XPS analyses were conducted in a 5600 multi technique ESCA/Auger (PHI, USA) at a base pressure ~2.5 × 10^−10^ mbar, incident monochromatic X-ray source (using Al K_α_ with energy 1486.6 eV) at a grazing 25° incidence angle, and spherical capacitor analyzer with slit aperture of 0.8 mm for analysis of the emitted photoelectrons. Helios G4 UC Dual Focus Ion Beam system from Thermo Fisher has been employed for secondary electron (SE) imaging with 2 keV electron beam and preparation of lamella for transmission electron microscopy (TEM) investigations. A Thermo Fisher Themis Z G3 TEM with a probe corrector for 300 keV electrons was employed for high-angle annular dark-field (HAADF) scanning TEM imaging and multiple (‘Super-X’) Silicon Drift Detectors for energy dispersive X-ray spectroscopy (EDS). Macro-magnetic properties were characterized using a superconducting quantum interference device (SQUID) magnetometer (MPMS-3 LOT-Quantum Design). The samples were measured using the vibrating sample magnetometry mode with peak amplitude of 4 mm and averaging time of 5 s. The data was corrected for remnant fields in a 7T-magnet and diamagnetic contribution of the Si substrate. 

## 3. Results and Discussion

### 3.1. Morphological Evolution upon Annealing

The morphological evolution of the as-deposited Py on the vicinal Si(111) substrate is evident through a series of topographic STM images and LEED patterns in Figure 1. The as-deposited Py film practically buried the substrate surface underneath, to the extent that the traces of the underlying Si(111)-(7×7) reconstructed terraces could be barely recognized in STM (Figure 1a,b), and only characteristically a faint (1×1) pattern on a milky background appeared in LEED (inset of Figure 1a), evidence of the amorphous nature of the coverage on top of now only bulk-terminated substrate. The (2 × 2)-reconstructed NDs began to form in the course of a 500 °C anneal, mostly by decorating the surface step-bunch ledges (Figure 1c,d). The milky background in the LEED pattern in the inset of Figure 1c was then replaced by mixed (2 × 2) + ($\sqrt{3}\times \sqrt{3}$) R30° superlattice diffraction spots (inset of Figure 1c), that became yet clearer and sharper (inset of Figure 1e) after further NDs growth by consumption of dilute Py (Figure 1e,f). We have previously witnessed the formation of a small fraction of ($\sqrt{3}\times \sqrt{3}$) R30°-reconstructed islands within the majority assembly of the (2 × 2)-reconstructed ones, as a reaction product of pure Fe with Si(111) [17]. 

### 3.2. XPS Study of the Nanodisk Bonding Chemistry 

**Figure 2:**
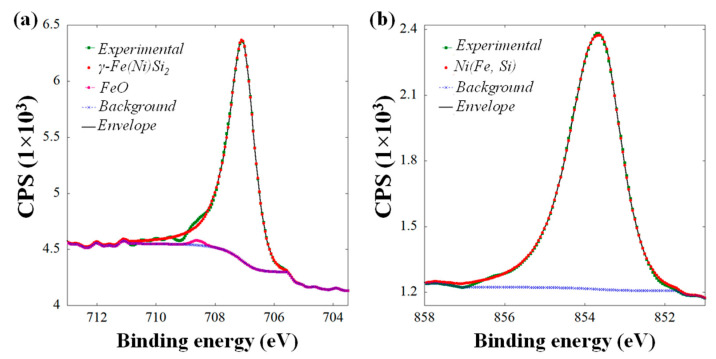
High resolution core level XPS spectra of (**a**) Fe 2*p*_3/2_ and (**b**) Ni 2*p*_3/2_ from the self-assembled NSs shown in Figure 1e.

Figure 2a,b represent the core level high resolution 2p_3/2_ XPS peak of Fe and Ni, respectively (shift corrected with respect to adventitious C 1s spectral position at 285 eV). Experimental data was analyzed using the Casa XPS software package [36] after background subtraction using Shirley’s method [37]. The relative quantification of several binding states of Fe 2p_3/2_ and Ni 2p_3/2,_ based on the integrated intensity of the corresponding spectral line has been detailed in Table 1. Using mixed Gaussian−Lorentzian, i.e., GL(m) (where m = 0 is a pure Gaussian and m = 100 is a pure Lorentzian) line shape modified by an asymmetric form, major peak of Fe 2p_3/2_ has been obtained at 707.08 eV. In our recent study, we reported the growth of γ FeSi_2_ phase on a (7 × 7) reconstructed Si(111) substrate with superlattice reconstruction (2 × 2) and ($\sqrt{3}\times \sqrt{3}$) R30° that corresponded to Fe 2p_3/2_ binding energy 707.2 eV [17,38]. The similarity of this line energy and shape pointed to the CaF_2_-based γ-phase nature of the present NDs, as well. Alongside, the minor peak at 708.59 eV fitted by line shape GL(0) matched well with FeO which was formed due to air exposure of our sample prior to XPS measurements [39,40]. In the case of Ni 2p_3/2_, fitting by GL(m) modified by exponential blend indicated only a peak at 853.61 eV. It is broadly accepted that the binding 2p_3/2_ energy of metallic Ni^0^ is 852.6 eV, with two main satellites located 3.8 eV and 6 eV above that [41,42,43]. However the 2p_3/2_ binding energy can rise above 853 eV in permalloys and other binary and ternary alloys and compounds [42,44,45], and in particular values close to those observed here of 853.61 eV, reflect the effect of the Si environment [46]. No significant presence of higher oxidation states of Ni^2+^ and Ni^3+^ oxides and hydroxides, situated at binding energies of 854 eV and above [47], could be detected in the Ni 2p_3/2_ line shown in Figure 2b. 

### 3.3. Structural and Compositional Analysis

**Figure 3:**
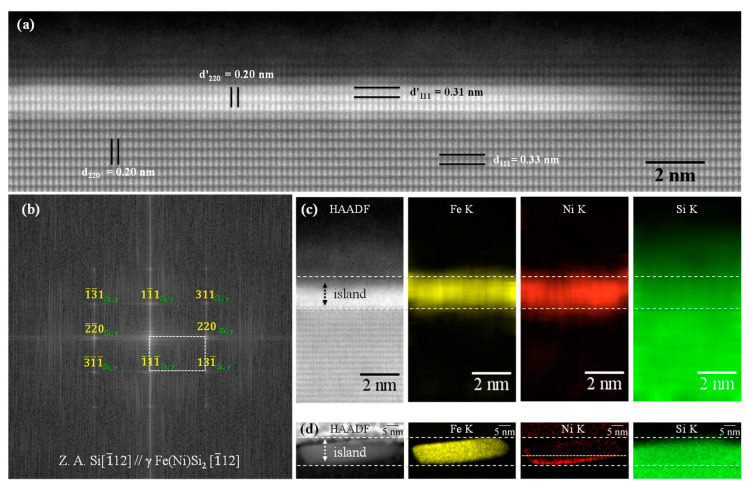
STEM analysis of typical γ-(Fe_x_Ni_1−x_)Si_2_ NSs: (**a**) cross-sectional HAADF micrograph, (**b**) corresponding Fourier power spectrum demonstrating [$\bar{1}12$]_Si_ || [$\bar{1}12$] _γ_ zone axis, and (**c**) HAADF and Fe-K, Ni-K, Si-K EDS maps. White broken lines in (**c**) outline the top and bottom γ-(Fe_x_Ni_1−x_)Si_2_ NS boundaries. (**d**) HAADF STEM micrograph and corresponding EDS elemental mapping of a representative β-Fe(Ni)Si_2_ phase NS given here for comparison, where white broken lines outline the top and bottom NS boundaries, and a yellow broken line roughly indicates the top boundary of the Ni-containing region close to the β-Fe(Ni)Si_2_/Si(111) interface.

The structural and compositional analysis of typical (2 × 2) reconstructed NSs was undertaken by cross-sectional TEM, as shown in Figure 3: HAADF micrograph (3a), and the corresponding Fourier power spectrum (3b), and elemental EDS mapping (3c). Previously, we reported flat-top γ-FeSi_2_ NSs on Si(001) [17] or flat-top β-Fe(Ni)Si_2_ NSs on Si(111) [35], that appeared to have only a disk shape in the top view, while in fact having faceted interfaces biting deep into the substrate. However, the pseudomorphic NSs grown in this work exhibited a genuinely disk-like shape, with flat top and bottom facets parallel to the Si(111) substrate surface, and atomically sharp, plane, and fully coherent interface (Figure 3a). Further, although caution must be exercised when defining the crystal structure of a 3–4 atomic layer thick NS, the real and Fourier space analysis of the NSs points to a CaF_2_-type crystal structure consistent with the γ-FeSi_2_ phase viewed along [$\bar{1}12$] zone axis, corroborated the conclusion of the XPS analysis above. The interplanar spacings of the Si substrate were marked as “d_hkl_” and of the NS as “d’_hkl_”, with measured d_111_ = 0.33 nm, d_220_ = 0.20 nm, and d’_111_ = 0.31 nm and d’_220_ = 0.20 nm, respectively.

Figure 3a,b indicates the following epitaxial orientation relations with substrate: {111}_Si_ ‖ {111}_γ_ and {110}_Si_ ‖ {110}_γ_. The EDS compositional mapping analysis revealed a uniform distribution of Fe, Ni and Si within the entire region of the NS (Figure 3c), signalling the formation of a truly ternary γ-(Fe_x_Ni_1−x_)Si_2_. We conclude that limiting the annealing temperature to 550 °C achieved the stated two-fold goal of staying below the temperature where (a) Ni in-diffusion and segregation towards the NS/Si interface practically renders the major top part of the β-NS devoid of Ni (as shown in Figure 3d, for comparison), and (b) γ → β transformation takes place [35]. 

### 3.4. Magnetometry 

**Figure 4:**
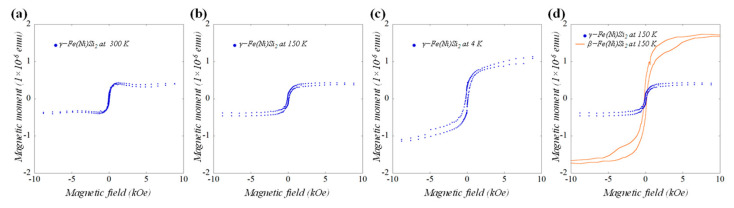
In-plane magnetization reversal loop of grown assembly of γ-Fe(Ni)Si_2_ NSs measured at (**a**) 300 K, (**b**) 150 K and (**c**) 4 K. Observed loop at 150 K for γ phase is compared with β phase in (**d**). The displayed magnetic moments have been normalized to reflect the twice larger volume of the as-deposited Py film in the γ-sample in comparison with that used to produce the β-sample in (**d**).

**Figure 5:**
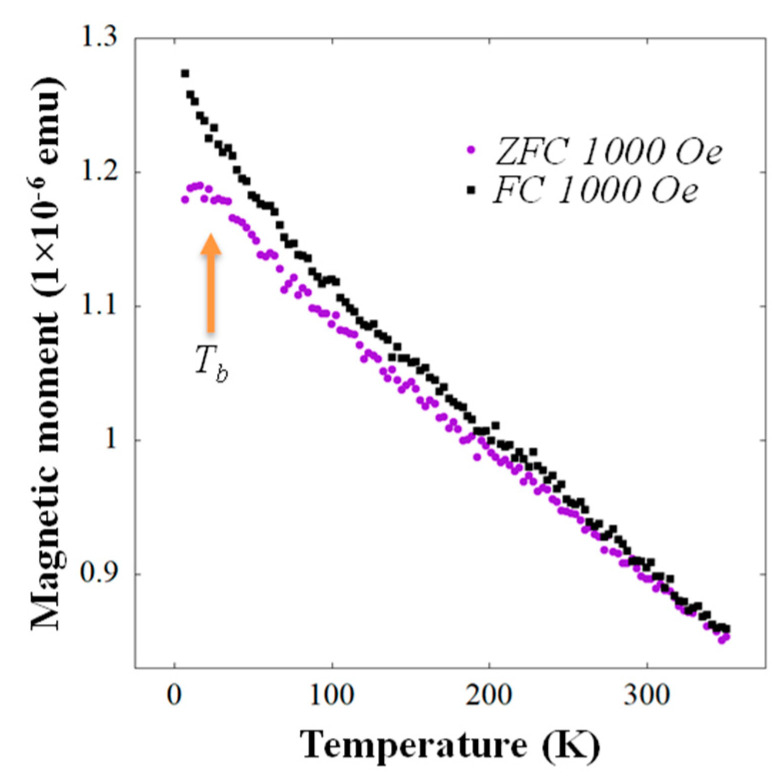
Temperature dependent, in-plane ZFC/FC study for assembly of the γ phase NSs with application of external magnetic field 1000 Oe.

To elucidate the magnetic properties of the self-assembled γ-(Fe_x_Ni_1−x_)Si_2_ NSs shown in Figure 1e,f, we acquired in-plane temperature-dependent magnetization reversal loops, and zero field cooled (ZFC) and field cooled (FC) curves. The systematic increase of the coercive field, saturation magnetic moment, and hysteresis loop area upon the measurement temperature lowering are apparent in Figure 4a–c. For example, the coercive field H_c_~72.4 Oe recorded at RT, increased to H_c_~104.2 Oe at 150 K, and to H_c_~166.0 Oe at 4 K (Figure 4a–c). The ZFC/FC curves indicate the bifurcation point temperature (T_irr_) in the vicinity of RT and a blocking temperature T_b_ at ~30 K (Figure 5), consistent with SPM behavior, where, however, transition to the blocked state for the largest NSs in the assembly takes place already at RT. The onset of transition to the blocked state for the NS distribution mean size was signified by T_b_. Such classical SPM behavior of noninteracting NSs is quite different from that in the presence of dipolar (namely SFM or SSG [17,19]) or direct exchange (e.g., ESM) interactions [35]. The magnetization reversal curve of the latter ESM was plotted up to scale, together with the present SPM one in Figure 4d for comparison (both measured at 150 K). Significant differences in the shape and opening of the hysteresis loop, as well as in the value of saturation magnetic moment, are apparent, most notably in the vicinity of the inflection points towards saturation magnetization. Absence of a clear bifurcation T_irr_ point, and a large temperature gap between the T_irr_ and T_b_, reflect the broad size and shape anisotropy distribution of the NSs in the assembly (cf. Figure 1c,e), and possibly the presence of two different (2 × 2) and ($\sqrt{3\;}\times \sqrt{3}$) R30° NS populations. 

## 4. Conclusions

The metastable CaF_2_-type γ-FeSi_2_ structure often precedes the thermodynamically stable α-FeSi_2_ and β-FeSi_2_ phases in thin epitaxial deposits. The purpose of this work was to exercise control over the sequence of phase formation and transformations in epitaxial silicide islands, with the aim of controlling the resulting physical properties of the so-formed nanosilicide arrays. In particular, we were interested in the magnetic properties of the reaction products of a soft Fe-Ni alloy (Ni_80_Fe_20_ Py, in this case) with Si, for a potential use in Si-based memory circuits and spintronic technology. Our previous experiments indicated that the ternary Fe-Ni-Si silicide phase transformation sequence followed the anticipated γ → β pattern, with, however, one significant difference—massive in-diffusion and segregation of Ni towards the interface with the Si substrate, resulting in a fascinating phenomenon of compositional separation of otherwise crystallographically identical β-phase into two magnetically different entities, whose coupling yielded an ESM behavior. 

Here, the aim was to form and stabilize ternary γ-disilicide islands and to avoid the loss of Ni, which was indeed achieved by limiting the maximum annealing temperature to 550 °C. On Si(111) surface, such a controlled heat treatment resulted in the formation of discrete compact ternary silicide NDs, with flat (2 × 2) or ($\sqrt{3\;}\times \sqrt{3}$) R30° reconstructed tops and fully coherent, pseudomorphic, and atomically sharp interfaces. 

Morphologically, although the islands were quite densely populating the preferential step-edge sites on the vicinal Si(111) surface, significant elongation into wires or long stripes did not eventually occur, leaving quite large gaps between the islands. Hence magnetically, formation of ESM was prevented by keeping the temperature low, and previously observed phenomena based on inter-island interactions, such as dipolar SSG or SFM, did not take place either. Instead, the SPM characteristic of a dilute ensemble of nanoparticles was apparent, with no clearly defined ZFC/FC bifurcation point and median blocking temperature around 30 K, reflecting the broad size, shape, and top-reconstruction distribution of the ternary γ-(Fe_x_Ni_1−x_)Si_2_ nanostructures with a small mean size. 

Control over the magnetic behavior of the nanoislands on Si surfaces through meticulous control over structural, morphological and compositional characteristics of their assembly, is imperative for applications in high-density data storage and spintronic devices.

## Figures and Tables

**Table 1:** Fitting parameters to high-resolution core level XPS spectra of Fe 2*p*_3/2_ and Ni 2*p*_3/2_ peaks (from Figure 2).

Phase	Binding Energy (eV)	FWHM (eV)	Line Shape	Concentration (%)
γ-Fe(Ni)Si_2_	707.08	0.92	A(0.35, 0.36, 0) GL(53.3333)	98.88
FeO	708.59	0.50	GL(0)	1.12
Ni(Fe, Si)	853.61	1.22	GL(67.6667) T(1.53)	100

## Data Availability

Data is contained within the present article.
